# MS-analysis of SILAC-labeled MYC-driven B lymphoma cells overexpressing miR-17-19b

**DOI:** 10.1016/j.dib.2016.02.031

**Published:** 2016-02-24

**Authors:** Marija Mihailovich, Tiziana Bonaldi

**Affiliations:** a Department of Experimental Oncology, European Institute of Oncology, Milan 20139, Italy

**Keywords:** MiR-17-92, SILAC, Quantitative proteomics, Mass spectrometry, MiRNA targets, MYC, B cell lymphoma

## Abstract

Micro RNAs (miRNAs) are small non-coding RNAs, which dampen gene expression by repressing translation and/or inducing degradation of target-mRNAs. Although the role of miR-17-19b (a truncated version of miR-17-92 cluster) is well documented in MYC-driven B cell lymphomagenesis, little is known about the function of the cluster in the maintenance of full-blown lymphomas. We employed SILAC-based quantitative proteomics to identify miR-17-19b targets upon a mild overexpression of the cluster in B cell lymphomas, established from λ-MYC transgenic mice. The proteomics data described in detail in this study, whose follow up analysis with MaxQuant algorithm is part of the recent publication (Mihailovich et al., 2015) [1], are deposited to the ProteomeXchange Consortium via the PRIDE partner repository, with the accession code PRIDE: PXD002810.

**Specifications table**

**Table:** 

Subject area	*Biology*
More specific subject area	*Proteomics*
Type of data	*Mass spectrometry raw data*
How data was acquired	*Data dependent LC-MS/MS acquired on* LTQ FT *(Thermo Fisher Scientific) coupled to a ultra nanoflow high-performance liquid chromatography (HPLC) system (EASY-nLC™ 1000, Thermo Fisher Scientific)*
Data format	*MS-RAW files*
Experimental factors	*Overexpression of miR-17-19b, a truncated version of miR-17-92 cluster, in full-blown B cell lymphoma from* λ*-MYC transgenic mice*
Experimental features	*Extracts from SILAC-labelled control and miRNA cells were in-solution trypsin-digested, separated by Isoelectro-focusing according to their isoelectric point (pI) and subjected to LC-MS/MS analysis.*
	*A 1:1 mixture of SILAC-labelled extracts from wild type (wt) versus wt cells was used as basal reference to set the cut-off values for the definition of the significant proteome response upon miRNA perturbation*
Data source location	*Milan, Italy, European Institute of Oncology*
Data accessibility	*Data described within this article and have been deposited to the ProteomeXchange Consortium via the PRIDE partner repository, with the dataset identifier PRIDE: PXD002810*

**Value of the data**

• Comprehensive high-confidence proteome of B lymphoma cells originated from λ-MYC transgenic mouse.
• Cellular response at the proteome level upon a mild induction of miR-17-19b cluster in full-blown B lymphoma cells.
• Possibility of using the dataset for the identification of miR-17-19b targets in a murine model of MYC-driven B cell lymphoma.
• Possibility of using the dataset for comparative analysis of different murine B cell lymphoma models.

## 1 Data

**Fig. 1:**
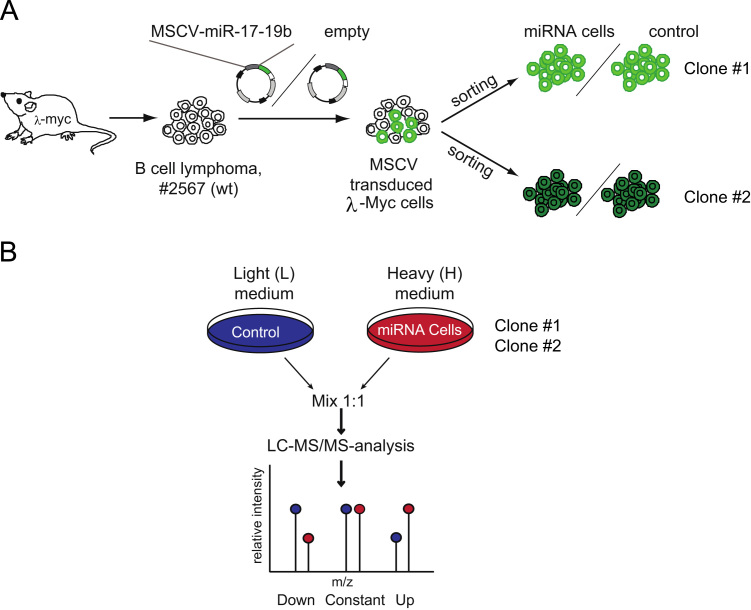
SILAC-based proteomic analysis of the B lymphoma response to miR-17-19b induction. (A) Scheme of the procedure for the generation of B lymphoma cells overexpressing miR-17-19b cluster (miRNA cells) or empty vector (control). (B) Scheme of the SILAC experiment in Dir setup.

Stable Isotope Labeling by Amino Acid in Cell culture (SILAC [2])-based quantitative proteomics was employed to analyze the impact of the miRNAs on the global protein output. In particular, we analyzed by high-resolution liquid chromatography coupled to tandem mass spectrometry (LC–MS/MS) full-blown B lymphoma cells, originated from λ-MYC transgenic mice [3], either with a mild overexpression of miR-17-19b (a truncated version of the miR-17-92 cluster), or -as a control- without overexpression of the cluster. In order to generate control and miRNA-overexpressing cells (miRNA cells), respectively, lymphoma cells were infected with a retroviral vector, empty or containing miR-17-19b (Fig. 1A).

The proteomics data obtained by the MS-analysis of these samples are described in the recent publication [1].

## 2 Experimental design, materials and methods

In the “Dir” SILAC experimental setups, miRNA cells were grown in Heavy (H) medium and control cells in Light (L) medium, whereas the SILAC channels were swapped in the Rev experiments. Light and Heavy cells were mixed 1:1, and the extracts were in-solution digested with trypsin. Sample complexity was reduced by peptide fractionation, using isoelectric focusing (IEF). LC–MS/MS was carried out on a LTQ-FT Ultra (Thermo Fisher Scientific). The data described hereby correspond to the SILAC-based quantitative analysis of functional experiments performed with two different clones (clones #1 and #2), and control experiment (wild type vs. wild type). See Table 1 and Fig. 1B.

### 2.1 Murine tumor cell line

Mouse B lymphoma cell line (clone ♯2567) was established through in vitro culture of primary lymphomas isolated from λ-MYC transgenic mice [3]. Tumors were cultured in B cell medium (DMEM medium (Invitrogen) supplemented with 10% fetal calf serum (FCS), 2 mM Glutamine (Lonza), 100 U/ml of penicillin and streptomycin (Lonza), 1 mM Na-pyruvate (Gibco), 1 mM non-essential amino acids (Gibco) and 50 mM β-mercaptoethanol (Gibco). Established IgM+B220+lymphoma cells were verified by Southern blot analysis for Igκ V gene rearrangements. IFOM animal Ethics Committee and the Italian Ministry of Health approved all procedures involving animals.

### 2.2 Cloning and retroviral gene transduction

The GFP containing expression vector pMIG was used for the generation of the plasmid pMIG-miR-17-19b. Fragment miR-17-19b, a truncated version of miR-17-92, was amplified by PCR from mouse genomic DNA (FW_BamHI_miR-17: 5’-CGG GAT CCG TCA GAA TAA TGT CAA AGT GCT-3’; RV_XhoI_miR-19b: 5’-CCG CTC GAG CAC TAC CAC AGT CAG TTT TGC AT-3’), and inserted into expression vector pMIG by BamHI-XhoI digestion. A spin infection of B lymphoma cells was performed with 2×10^5^ cells per well. Retroviruses were produced by Phoenix cells, which were previously transfected with pMIG and pMIG-miR-17-19b plasmids. The efficiency of transduction was evaluated by flow cytometric analysis for GFP positive cells 48 h post-infection, and subsequently GFP-sorted. We performed two independent rounds of sorting for control and miRNA cells, obtaining two different cell populations, which we refer to as clones #1 and #2. The experiments have been performed within the first ten cell passages post-infection.

### 2.3 SILAC labeling

Control and miRNA cells were grown in SILAC media (lysine- and arginine-free DMEM/Ham’s F12 (1:1), 10% dialyzed fetal bovine serum (FBS, Invitrogen)), supplemented with 1 mM non-essential amino acids (Gibco), 100 U/ml of penicillin and streptomycin (Lonza), 1 mM Na-pyruvate (Gibco), 2 mM Glutamine (Lonza) and 50 mM β-mercaptoethanol (Gibco)). “Heavy” and “Light” media were obtained by adding 0.146 g/L ^13^C_6_, ^15^N_2_ L-Lysine and 0.84 g/L ^13^C_6_^15^N_4_ L-Arginine (Sigma) or the corresponding non-labeled amino acids, respectively to the SILAC media. Growth in SILAC media was carried out for eight duplications, to ensure complete protein labeling. For the Dir experiments miRNA cells were labeled with heavy medium, and control cells with light medium, while for the Rev experiment, we grew control cells in heavy, and miRNA cells in light medium.

### 2.4 In solution digestion and isoelectro-focusing of peptide mixtures from the functional experiments (control:miRNA cells)

Equal numbers (12×10^6^) of heavy and light cells were mixed and lysed in 300 ⎕l RIPA buffer (10 mM Tris–HCl, pH 8.0, 1% Triton, 0.1% SDS, 0.1% Deoxycholate, 140 mM NaCl, 1 mM EDTA, 1 mM DTT, 1 mM PMSF and protease inhibitor cocktail (Roche)). Total protein extracts (150 μg) from 1:1 mix of labeled control and miRNA cells were dissolved in denaturation solution (6 M urea, 2 M thiourea in 20 mM ammonium hydrogen carbonate) for 30 min. After complete denaturation, extracts were supplemented with 1 mM DTT and incubated for additional 30 min. Thyols were carboxymethylated with 5 mM IAA for 20 min. Proteins were first digested with 1 μg LysC for 3 h, then diluted 4× with 50 mM ammonium bi-carbonate, and digested with 1 μg trypsin overnight at 37 °C. The digestion was stopped by acidifying the sample to pH<2 with 100% TFA. We separated peptides according to their isoelectric point (pI) by isoelectrofocusing electrophoresis, using the Agilent 3100 OFFGEL Fractionation Kit (Agilent Technologies) [4], according to the manufacturer’s protocol. After separation, peptides mixtures focused at different pH were reconstituted with 1% TFA and desalted and purified on C_18_ STAGE tips [5].

### 2.5 In gel digestion of the wt:wt extracts

Protein samples were separated on a 8–12% gradient mini gel (Invitrogen). After Coomassie staining (Colloidal Blue Staining Kit, Invitrogen), each line was cut in fifteen slices and trypsin digested according to a previously described protocol [6]. Briefly, after distaining with 50% acetonitrile (ACN)/25 mM ammonium bicarbonate (NH_4_HCO_3_) solution, and dehydration by 100% ACN, gel pieces were incubated with 10 mM dithiothreitol in 50 mM NH_4_HCO_3_ for 60 min at 56 °C for cysteine reduction and then alkylated with 55 mM iodoacetamide in 50 mM NH_4_HCO_3_ for 45 min at room temperature, in dark. After several rounds of washings with 50 mM NH_4_HCO_3_ and dehydration with 100% ACN, proteins were digested with trypsin overnight, at 37 °C. The reaction was stopped by acidification with 2 μl of 50% trifluoroacetic acid (TFA). Peptides were eluted with 30% ACN/3% TFA and 100% ACN. After speed-vacuum centrifugation, peptides were solubilized in 100 μl of 0.1% formic acid (FA), desalted and concentrated using reverse phase C_18_ Stage Tips [5]. Peptides were eluted with 80% ACN, lyophilized and re-suspended in 7 μl of 0.1% formic acid for LC–MS/MS analysis.

### 2.6 LC–MS/MS

Peptide mixtures, eluted from C_18_ Stage tips, were separated by reversed-phase chromatography on an in-house-made 15 cm column (outer diameter 350 μm, inner diameter 75 μm, 1.9 μm ReproSil, Pur C_18_AQ medium), using a ultra nanoflow high-performance liquid chromatography (HPLC) system (Agilent 1100 Series nano-flow LC system (Agilent Technologies)), directly interfaced to a LTQ-FT Ultra mass spectrometer (Thermo Fisher Scientific). The composition of solvent A was 0.1% FA and 5% ACN in H_2_O and of solvent B was 95% ACN with 0.1% FA. Samples were injected in 0.1% TFA solution at a flow rate of 500 nl/min. The gradient was 140 min, starting from 2% till 60% ACN in 0.5% acetic acid. The spray voltage of a nanoelectrospray ion source (Proxeon) was 1.5–2.0 kV; the temperature was set to 180 °C. We used data-dependent mode to automatically switch between MS and MS/MS acquisition. The full scan MS spectra were acquired at a target value of 2,000,000 ions and with a resolution of 100,000 (FWHM) at 400 m/z, while MS/MS spectra were acquired using a target value of 5000 ions and the five most intense ions were isolated for CID-fragmentation.

## Figures and Tables

**Table 1:** List and description of all LC-MS/MS data acquired.

	File name	format	preparation	experimental setup	sample
1	F090801_EV_BCELL_PMIG1719_Dir1	24 wells	in solution digestion+off-gel	Dir1	Clone #1
2	F091104_EV_BCells_mig(17-19)1to1	24 wells	in solution digestion+off-gel	Dir1 bis	Clone #1
3	F090804_EV_Bcell_HL_Rev1	24 wells	in solution digestion+off-gel	Rev1	Clone #1
4	F090925_EV_Bcell_HL_Rev1bis	24 wells	in solution digestion+off-gel	Rev1 bis	Clone #1
5	F090807_EV_BCELL_PMIG1719_Dir2	24 wells	in solution digestion+off-gel	Dir2	Clone #1
6	F091127_EV_BCell_Dirb2b	24 wells	in solution digestion+off-gel	Dir2 bis	Clone #1
7	F101215_EV_BCell_HL1to1_Dir2_Sort3	24 wells	in solution digestion+off-gel	Dir2	Clone #2
8	F101217_EV_Bcell_HL1to1_Rev1_Sort3	24 wells	in solution digestion+off-gel	Rev1	Clone #2
9	F090303_EV_2567BcellHL1to1_A	15 slices	in gel digestin	NA^b^	wt:wt
10	F090303_EV_2567BcellHL1to1_B	15 slices	in gel digestin	NA	wt:wt
11	F090401_ev_Bcell_HL1to1	15 slices	in gel digestin	NA	wt:wt
12	F080904TB_yeast_2567^a^	15 slices	in gel digestin	NA	wt:wt
13	F080917TB_2567: 15 row files in duplicate	15 slices	in gel digestin	NA	wt:wt

a The name of the file refers to the fact that this sample was run after a yeast quality control sample; no yeast extract is anyhow present in any of the samples used in this study.

b In the wt:wt experiments (9–13), heavy and light cell populations are the same, therefore the definition of the experimental setup Dir and Rev is not applicable.
